# The Effect of MCP-1/CCR2 on the Proliferation and Senescence of Epidermal Constituent Cells in Solar Lentigo

**DOI:** 10.3390/ijms17060948

**Published:** 2016-06-15

**Authors:** Woo Jin Lee, Soo Youn Jo, Mi Hye Lee, Chong Hyun Won, Mi Woo Lee, Jee Ho Choi, Sung Eun Chang

**Affiliations:** Department of Dermatology, Asan Medical Center, University of Ulsan College of Medicine, Seoul 138-736, Korea; uucm79@hanmail.net (W.J.L.); sooyounyda@gmail.com (S.Y.J.); algp_@naver.com (M.H.L.); chwon98@chol.com (C.H.W.); miumiu@amc.seoul.kr (M.W.L.); jhchoy@amc.seoul.kr (J.H.C.)

**Keywords:** solar lentigo, monocyte chemoattractant protein-1, epidermal constituent cells

## Abstract

Solar lentigo (SL) is a representative photoaging skin disorder. Alteration of the main epidermal constituent cells—keratinocytes and melanocytes—in relation to the photoaged dermal environment or chemokine/cytokine network is suggested as its pathogenesis. Among these, we focused on monocyte chemoattractant protein-1 (MCP-1), as it is known to be associated with tissue aging. For the first time, we report that the MCP-1 receptor, CCR2, is expressed in normal human melanocytes. In SL tissue, there was an increase of CCR2+Melan A+ melanocytes with positivity to Rb protein compared to peri-lesional normal skin. MCP-1 induced the proliferation of normal human melanocytes without a significant change in the melanin content. MCP-1 treatment in normal human keratinocytes showed an increase in senescence-associated β-galactosidase staining and p53 and p21 protein expressions. In summary, MCP-1 may participate in the development of SL by affecting epidermal constituent cells, for example, by inducing melanocyte proliferation and keratinocyte senescence.

## 1. Introduction

Solar lentigo (SL) or senile lentigo is a very common hyperpigmentary skin disorder that is caused by chronic exposure to ultraviolet radiations (UVR). SL lesions are disfiguring especially on the face; thus, melanosome-selective Q-switched lasers are often used in the treatment of SL [1]. However, inflammatory events and/or recurrence are often observed with age in SL [1,2]. Even before laser treatment, SL lesion is associated with an increase in inflammatory and dendritic cells that release various inflammatory mediators stimulated by UVR [3]; these inflammatory features are also seen in other common photoaging skin disorders such as melasma [4]. Photoaging-associated benign lichenoid keratosis, which is characterized by a flattening of the epidermis with lichenoid infiltration of dermal inflammatory cells, can be developed in the SL lesion [5]. Interestingly, dermal factors secreted by irradiated aged fibroblasts were demonstrated to induce SL in pigmented reconstructed epidermis [6].

Histologically, SL is characterized by a hyperpigmented basal layer, often with a thickened epidermis showing elongated, clubbed rete ridges, usually with an increased number of melanocytes [3,7]. However, the pathogenesis of SL development is far from being completely understood, even though the accelerated differentiation of melanocyte stem cells has been suggested [8,9].

Melanocytes are actively involved in the paracrine network of the cytokine-chemokine system, interacting with epidermal keratinocytes and the dermal compartment cells [10,11]. We hypothesized that a histopathological alteration of the main constituent epidermal cells—keratinocytes and melanocytes—may be related to the dermal environment of the chemokine/cytokine network in SL pathogenesis.

Among these, we focused on the role of monocyte chemoattractant protein-1 (MCP-1) in SL, as it is a well-known chemokine associated with the tissue aging phenotype [12] and has not been investigated in human melanocyte biology. Senescent cells play an important role in modulating the microenvironment by secreting biologically active molecules such as cytokines (interleukin (IL)-6, IL-1) and chemokines (CXCL8/IL-8, CCL2/MCP-1), thus giving rise to senescence-associated secretory phenotype (SASP) [13,14]. MCP-1 is a proinflammatory chemokine that participates in the recruitment of monocytes and lymphocytes [15]. In the skin, MCP-1 is expressed and produced by inflammatory cells, stromal cells, and keratinocytes [16,17]. SASP in stromal cells is reported to contribute to the establishment of epithelial cell senescence in adjacent tissues [18]. MCP-1, one of the most well-known chemokines of the SASP, is released in the skin upon exposure to UVR [19].

No study has yet examined MCP-1 and its receptor, CCR2, in normal human melanocytes (NHMs) or the effect of MCP-1/CCR2 on keratinocytes and melanocytes, which comprise the main constituent cells in SL. Here, we investigated whether MCP-1/CCR-2 affects the cellular activity or senescence in melanocytes and keratinocytes *in vitro* and in skin tissues from SL lesions.

## 2. Results

### 2.1. Increment of Melanocytes in Solar Lentigo (SL) Tissue

**Figure 1:**
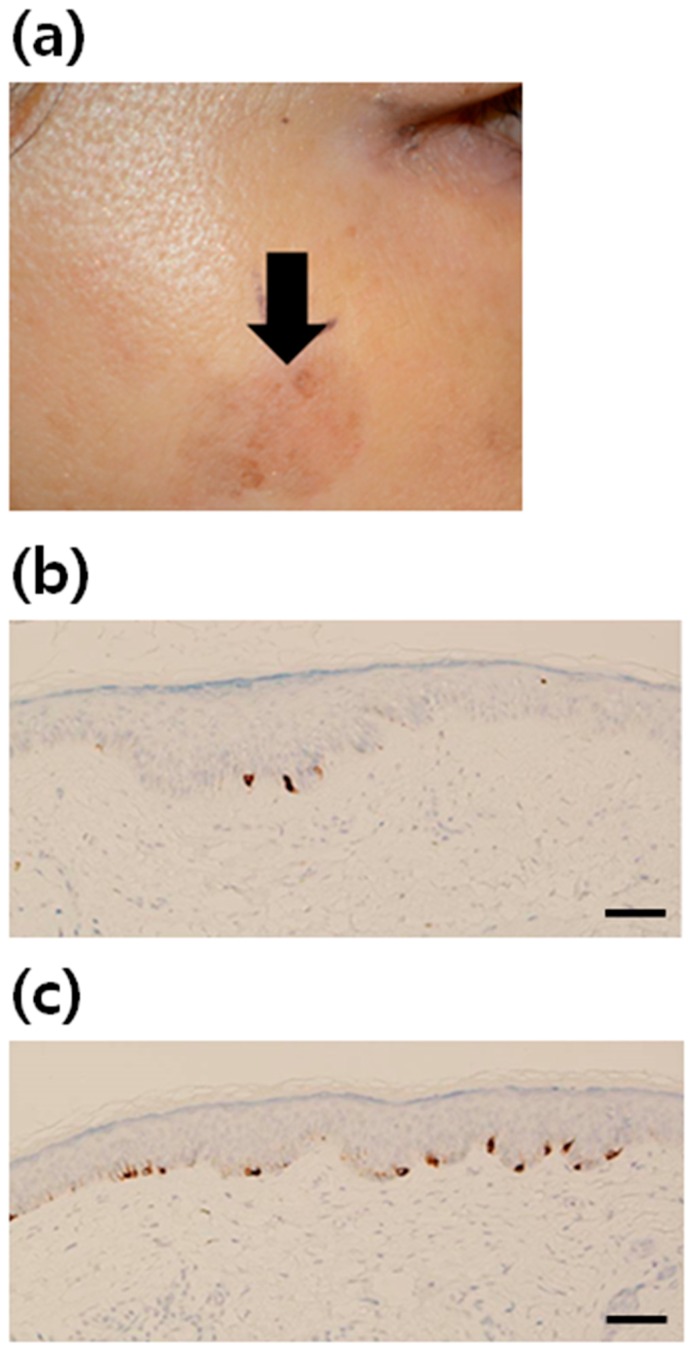
Increment of melanocytes in solar lentigo (SL) tissue. (**a**) A clinical photograph of facial SL. Immunohistochemical staining using antibodies to Melan A (brown color) in (**b**) normal skin (×200) and (**c**) SL (×200). The number of melanocytes was increased in the lesional skin of SL, compared with that in the non-lesional skin (scale bar: 50 µm).

SL tissue samples from a total of 21 healthy Korean women were studied (Table 1). As the ratio of melanocytes to keratinocytes is known to be 0.13~0.20 in the normal skin, depending on the linear keratinocyte density (per mm), we considered the ratio of keratinocytes to melanocytes under 5~6:1 as an increase in melanocytes from case to case [20]. Increment of melanocytes was found in 62% of the patients. The number of Melan A-positive melanocytes appeared to be increased in the lesional skin of SL, compared to that in the non-lesional skin (Figure 1)

### 2.2. Expression of CCR2 in Normal Human Melanocytes (NHMs) and SL Skin Tissue 

**Figure 2:**
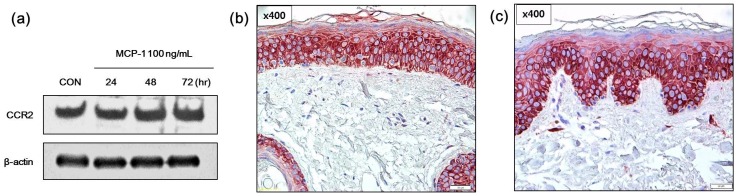
Effects of monocyte chemoattractant protein-1 (MCP-1) on CCR2 expression in (**a**) normal human melanocytes (NHMs) were evaluated by Western blotting. Cells were treated with 100 ng/mL MCP-1 for the indicated times. Cell lysates were assessed by Western blot analysis using antibodies against CCR2. β-actin was used as the loading control. Immunohistochemical analysis of CCR2 expression in (**b**) normal skin tissue and (**c**) SL tissue (scale bar: 50 µm). In both normal skin tissue and SL tissue, CCR2 staining was observed in the epidermal constituent cells including keratinocytes and melanocytes.

First, the presence of the MCP-1 receptor, CCR2, in melanocytes was examined *in vitro* as well as in normal skin and SL tissues. CCR2 expression following treatment with 100 ng/mL of MCP-1 for 24–72 h was observed in NHMs (Figure 2a). CCR2 was expression was also evaluated in normal skin and SL skin tissues by immunohistochemical analysis. CCR2 was positive in the cytoplasm of both normal skin tissue (Figure 2b) and SL tissue (Figure 2c). These findings indicate that MCP-1 functions in the skin tissue through CCR2.

### 2.3. Cell Viability and Proliferation after MCP-1 Treatment in Normal Human Keratinocytes (NHKs) and NHMs

**Figure 3:**
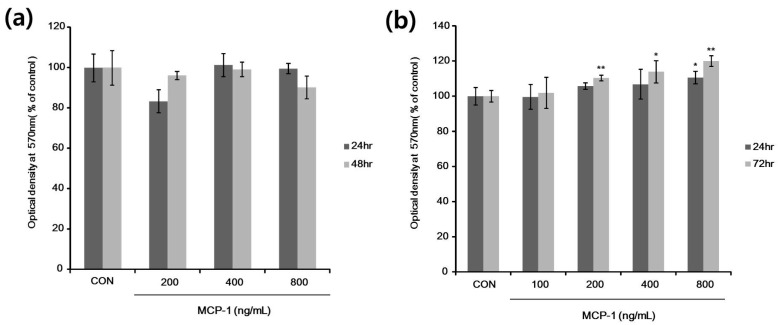
*In vitro* cell proliferation evaluated using the 3-(4,5-dimethylthiazol-2-yl)-2,5-diphenyl tetrazolium bromide (MTT) assay. (**a**) Normal human keratinocytes (NHKs) were treated with 200–800 ng/mL MCP-1 for the indicated times; (**b**) NHMs were treated with 100–800 ng/mL MCP-1 for the indicated times, followed by addition of MTT, and absorbance was then measured at 570 nm in a microplate reader. * *p* < 0.05, and ** *p* < 0.01 by the Student‘s *t*-test.

To determine the experimental concentration of MCP-1, MCP-1 cytotoxicity was evaluated using the 3-(4,5-dimethylthiazol-2-yl)-2,5-diphenyl tetrazolium bromide (MTT) cell proliferation assay. According to the results, 200–800 ng/mL MCP-1 caused no significant changes in NHK viability (Figure 3a); we therefore used these concentrations in NHK experiments. NHMs were incubated in the presence of MCP-1 at doses of 100–800 ng/mL for 24 or 72 h. As shown in Figure 3b, the proliferation of NHMs was observed at an MCP-1 dose of 200 ng/mL. NHM proliferation was increased after treatment with MCP-1 in a dose- and time-dependent manner.

### 2.4. Morphological Changes and Senescence-Associated Beta-Galactosidase (SA-β-Gal) Staining in NHKs

**Figure 4:**
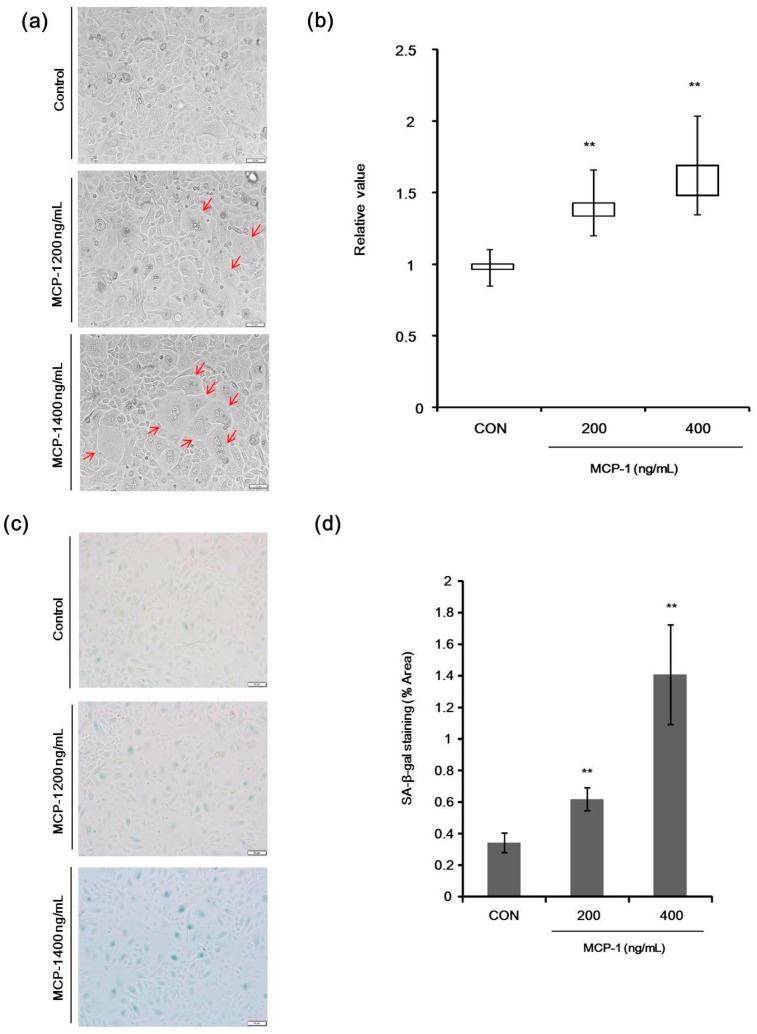
(**a**) Morphological changes in NHKs following treatment with MCP-1 (200 or 400 ng/mL) were observed (arrow) (scale bar: 50 µm); (**b**) nuclear length was calculated using the Image J software on bright field microscope image. Three fields were selected to calculate nuclear size (10 cells per field). Median, interquartile range, minimum, and maximum are depicted by box plots; (**c**) effects of MCP-1 on NHK senescence were evaluated by Senescence-Associated Beta-Galactosidase (SA-β-Gal) staining. NHKs were seeded at the same density in 6-well plates and were stained for SA-β-Gal, using a commercial histochemical staining kit. SA-β-Gal activity (blue lights) was increased in the presence of 200–400 ng/mL MCP-1 for 72 h (scale bar: 50 µm); (**d**) five fields were selected to calculate the SA-β-Gal staining area in each group and the average was taken as the total area. The data are presented as the mean ± SD (*n* = 3). ** *p* < 0.01 by the Student’s *t*-test.

Significant morphological changes were observed in NHKs following treatment with MCP-1 (Figure 4a). Nuclear size enlargement, an established marker of *in vitro* senescence was observed in cultured NHKs when treated with MCP-1 (Figure 4b). The effects of 200–400 ng/mL MCP-1 on NHK senescence were detected by Senescence-Associated Beta-Galactosidase (SA-β-Gal) staining (Figure 4c). SA-β-Gal activity was increased in a dose-dependent manner in NHKs following MCP-1 treatment (Figure 4d).

### 2.5. Senescence-Associated Markers in NHKs

**Figure 5:**
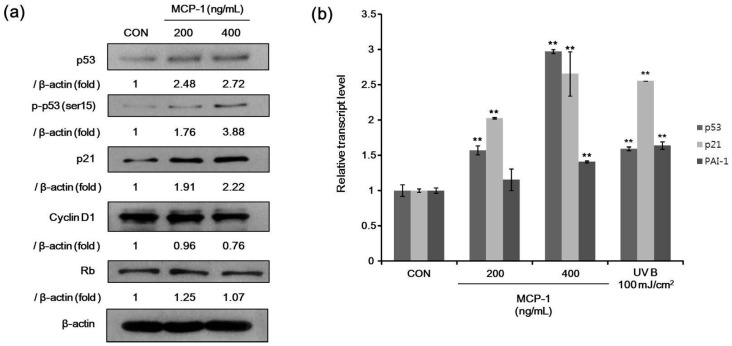
(**a**) The expression level of senescence-associated proteins in NHKs following MCP-1 treatment for 72 h was examined by Western blotting analysis. β-actin was used as the loading control; (**b**) qPCR analysis detected a induction of mRNA levels of p53 and p53 target genes p21 and plasminogen activator inhibitor-1 (PAI-1) in NHKs following MCP-1 treatment for 48 h. UV irradiation was used as positive control. The data are presented as the mean ± SD (*n* = 3). ** *p* < 0.01 by the Student’s *t*-test.

For senescence markers, we determined the protein levels of p53, phosphorylated p53 (Ser 15), Rb, p21, and cyclin D1 after treatment with MCP-1 in NHKs. The expression levels of p53, phosphorylated p53, and p21 were increased, while cyclin D1 was decreased when NHKs were treated with MCP-1 (Figure 5a). Expression levels of three genes involved in cellular senescence were determined in NHKs. We found an increased gene expression of p53, p21, and plasminogen activator inhibitor-1 (PAI-1) upon MCP-1 treatment (Figure 5b). UV irradiation was used as a positive control and also increased the expression of these analyzed genes. These suggested that MCP-1 induces cellular senescence in NHKs. These findings indicate that MCP-1 treatment in NHKs induces growth inhibition and senescence.

### 2.6. Melanin Contents and SA-β-Gal Staining after Treatment with MCP-1 in NHMs

**Figure 6:**
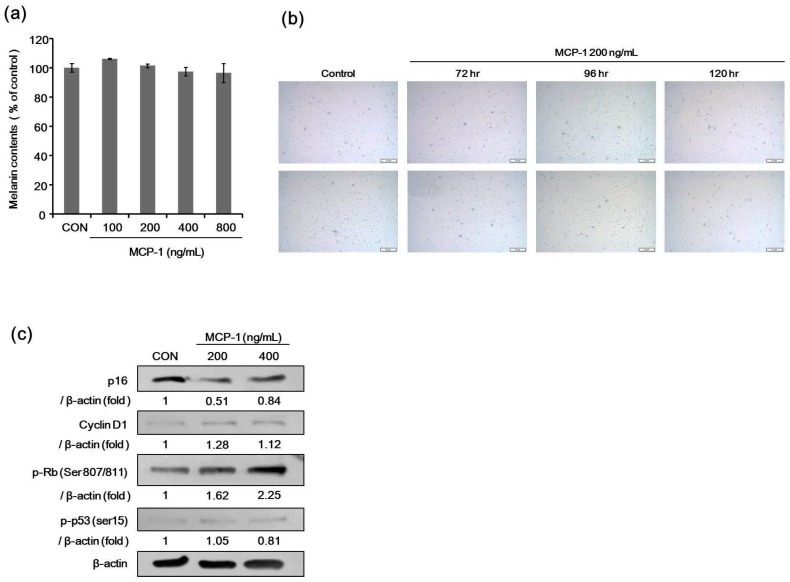
(**a**) Effects of MCP-1 on melanogenesis. Melanin content was measured in NHMs after 120 h of treatment with 100–800 ng/mL MCP-1. Data are the mean ± standard deviation (SD) of triplicate assays and are expressed as a percentage of the control; (**b**) the effects of MCP-1 on NHM senescence were evaluated by SA-β-Gal staining. Cells were seeded at the same density in 6-well plates and were stained for SA-β-Gal using a commercial histochemical staining kit (scale bar: 50 µm); (**c**) proliferation-associated proteins in NHMs were determined after treatment with 200 or 400 ng/mL MCP-1 for 72 h. β-actin expression was detected as the loading control.

We determined the melanin content in melanocytes after treatment with MCP-1. There was no significant difference in melanin content after MCP-1 treatment for 120 h at 100–800 ng/mL (Figure 6a). The effects of MCP-1 on melanocyte senescence were evaluated by SA-β-Gal staining. There was no significant change in SA-β-Gal activity after MCP-1 treatment in melanocytes (Figure 6b).

### 2.7. Expression of Proliferation Markers in NHMs Treated with MCP-1

The expression level of phosphorylated Rb and cyclin D1 increased, but the level of p16 and phosphorylated p53 were decreased when NHMs (Figure 6c) were treated with 200 or 400 ng/mL MCP-1 for 72 h. These findings indicate that MCP-1 induces CCR2 activation and cellular proliferation in melanocytes.

### 2.8. Intra-Epidermal Distribution of CCR2 and Rb in SL Tissue Evaluated by Confocal Laser Microscopy

**Figure 7:**
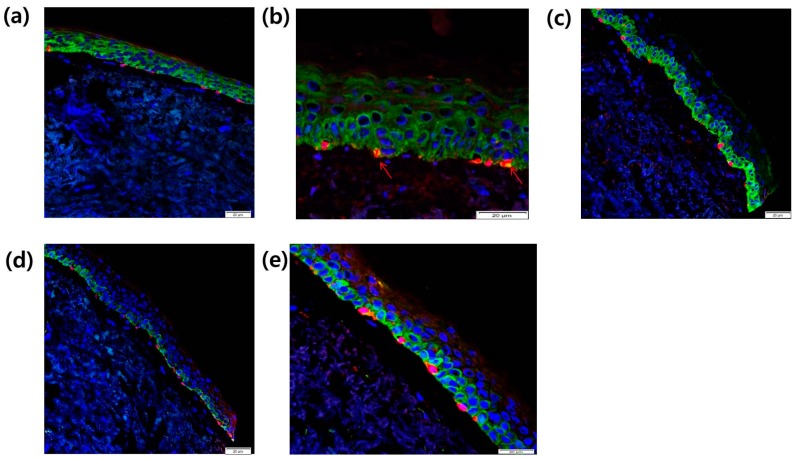
Localization of CCR2 in the epidermis from (**a**) normal skin tissue and (**b**) SL tissue was evaluated by double immunofluorescence staining. Skin tissues were stained with Alexa-Fluor 546-conjugated (for Melan A (red)) secondary antibody for 30 min. The samples were washed, and then incubated with FITC-conjugated goat anti-rabbit IgG secondary antibody against Rb and CCR2 (green) for 30 min at room temperature (scale bar: 20 µm). Yellow and arrow, Melan A-positive melanocytes with CCR2 co-localization; (**c**–**e**) After DAPI staining, the sections were mounted and the localization of Rb (green), CCR2, and Melan A (red) was detected using a confocal laser scanning microscope (scale bar: 20 µm).

To investigate the localization of CCR2 in melanocytes from SL tissue, we used immunohistochemical staining with antibodies specific to these proteins to compare their distribution between normal tissue and SL lesional tissue by confocal laser microscopy. CCR2 localization (green) in the epidermis was found both in normal skin tissue (Figure 7a) and in SL tissue (Figure 7b). In SL tissue, increased Melan A-positive melanocytes (red) with CCR2 co-localization (yellow, arrow) were found along the basal layer in the epidermis (Figure 7b). We also investigated the localization of the proliferation marker Rb in melanocytes from SL tissues. Rb expression (green) in the epidermis and Melan A-positive melanocytes (red) were found to be increased in the SL tissue (Figure 7c) compared to the normal skin tissue (Figure 7d). In lentigo tissue, co-localization of Melan A (red) and Rb (green) was noted in the basal melanocytes (Figure 7e).

## 3. Discussion

MCP-1 (CCL2) is a member of a subfamily of the IL-8 supergene family, which is a proinflammatory chemokine that participates in the recruitment of monocytes and lymphocytes [15]. In the skin, MCP-1 is expressed and produced by inflammatory and stromal cells such as fibroblasts and endothelial cells, and its expression is upregulated following proinflammatory stimuli and tissue injury [16,17,21]. UV exposure has been associated with the release of various inflammatory mediators that are involved in SL pathogenesis [6,22,23]. Melanocytes are actively involved in the paracrine network of the cytokine-chemokine system and neuroendocrine network, interacting with epidermal keratinocytes and the dermal compartment cells [11,24,25,26]. UVR and immune cytokine regulate the cutaneous levels of proopiomelanocortin (POMC) and corticotropin releasing hormone (CRH) [27]. CRH can be released from skin sensory nerves and immune cells in response to environmental changes [27]. CRH stimulates production of POMC with increased activation of adrenocorticotropic hormone (ACTH) and α-melanocyte-stimulating hormone (MSH) [28]. Melanogenesis is controlled by highly structured systems including hormonal regulation and nutritional factors [29,30].

MCP-1 mediates its effects through its receptor CCR2. Unlike MCP-1, CCR2 expression is relatively restricted to certain types of cells [12]. Herein, we demonstrated for the first time that CCR2 is present in NHMs *in vitro* and in skin tissues. Although the role of MCP-1 in NHM biology is not known, MCP-1 potentiates tumor necrosis factor-α (TNF-α) and IL-1α in the stroma, and may potentiate early tumor growth and tumor angiogenesis in malignant melanoma [31]. Production of MCP-1 from non-pigmented mouse melanocytes has also been reported in [32], wherein the authors suggested that MCP-1 contributes to tumor angiogenesis [32].

Our histological evaluations of SL lesions in elderly women showed an increased number of melanocytes even in the absence of rete ridge elongation. Previous studies have also indicated an increased number of melanocytes in SL [3,7]. In the present study, SL lesions showed increased expression of CCR2+Melan A+ melanocytes in the lesional epidermis with Rb positivity. As treatment with MCP-1 induced proliferation of NHMs *in vitro* and increased the CCR2+ melanocytes in SL, it can be postulated that the paracrine effect of MCP-1 could induce melanocyte proliferation in SL. In this case, MCP-1 might be released by the photoaged dermal compartment composed of inflammatory cells and stromal cells of SL in the lesional epidermis. There are evidences that the UVR-induced dermal inflammatory reaction or keratinocytic responses induce melanocyte proliferation via the cytokines/chemokines system, and that chemokines are associated with melanocyte migration [2,22,23,24].

In our study, MCP-1 alone had no significant effect on melanogenesis. It is postulated that MCP-1 and other inflammatory cytokines such as TNF-α and IL-1α may upregulate melanogenesis via a paracrine network involving the epidermal and dermal compartment cells in the cellular environment of SL [10,11,22]. Increased melanogenic potential of melanocyte in SL lesions may not be solely attributed to the paracrine effect of chemokines, but be associated with multifactorial pathway such as accelerated melanocyte stem cells induced by Wnt1 expression [8].

MCP-1 relays and reinforces senescence signaling by increasing the protein levels of p53 and p21 via ROS or p38 MAPK signaling [33,34]. MCP-1 has a positive feedback with inherent senescence processes in mesenchymal stromal cells [33]. In our *in vitro* experiment, as expected, treatment with MCP-1 induced senescence in NHKs. SASP regulates cellular senescence through paracrine or autocrine mechanisms [34]. SASP produced by dermal fibroblasts was suggested to be associated with intrinsic skin aging [18]. SL is a representative manifestation of photoaging, but SASP related to skin photoaging has not been elucidated. In one previous study, UVB-exposed keratinocytes were found to produce MCP-1 [19]. We believe that MCP-1/CCR2 might have a role in the senescence of keratinocytes during the development of SL as an autocrine effect from keratinocytes or as a paracrine effect from the dermal compartment.

In summary, we suggest that MCP-1/CCR2 affects the proliferation and senescence of constituent epidermal cells in relation to SL development.

## 4. Materials and Methods

### 4.1. Reagent and Antibodies

Recombinant human CCL2/MCP-1 (279-MC/CF-050) was obtained from R&D Systems (Minneapolis, MN, USA). The antibody specific for beta-actin was purchased from Sigma-Aldrich (St. Louis, MO, USA). The antibody specific for CCR2 (ab32144) was purchased from Abcam (Cambridge, UK). Antibodies specific for total p53 (1C12) (#2524), Phospho-p53 (ser15) (#9284), and Phospho-Rb (Ser807/811) (#9308) were purchased from Cell Signaling Technology (Beverly, MA, USA). The antibody specific for total Rb (C-15) (sc-50) and p16 (JC8) (sc-56330) were purchased from Santa Cruz Biotechnology, Inc. (Santa Cruz, CA, USA). The antibodies specific for P21 (GTX27960) and Cyclin D1 (GTX112874) were purchased from GeneTex, Inc. (San Antonio, TX, USA). The antibody specific for MART-1 (Melan A) (A103) was purchased from Cell Marque Co. (Rocklin, CA, USA).

### 4.2. Cell Culture

Cultured NHMs and NHKs were purchased from Invitrogen (Carlsbad, CA, USA). We used cells at passages between 3 and 6 in our experiments. NHMs were maintained in Medium 254 (Cascade Biologics, Portland, OR, USA) containing human melanocyte growth supplement at 37 °C in 5% CO_2_. NHK cells were cultured in EpiLife^®^ Medium and keratinocyte growth supplements (Cascade Biologics). Cells with densities of 5 × 10^3^ for each well were placed in 96-well plates at 37 °C in 5% CO_2_.

### 4.3. Cell Proliferation Assay

NHKs and NHMs were seeded at the same density in a 96-well plate. MTT (Duchefa, Haarlem, Netherlands) was prepared as a 2.5 mg/mL stock solution in phosphate-buffered saline (PBS) and stored at 4 °C. Next, stock MTT solution was added at 10 μL/well, and the plates were incubated at 37 °C for 2 h. MTT solution was removed, 100 μL of DMSO was added to the plates, and the plates were incubated for 15 min at 37 °C to dissolve the formazan crystals. Absorbance was measured at 570 nm using an enzyme immunosorbent assay (ELISA) reader (Molecular Devices Co., Sunnyvale, CA, USA). The reference wavelength was 560–650 nm.

### 4.4. Melanin Content Assay

NHMs were seeded onto 6-well tissue culture plates at densities of 6 × 10^5^ cells/well. Cells were treated with MCP-1 in Medium 254 containing human melanocyte growth supplement for 5 days. The cells were then dissolved in 550 μL of 1 N NaOH at 100 °C for 30 min and centrifuged at 13,000 rpm for 5 min. The optical density (OD) of the supernatant was measured at 405 nm in a microplate reader. Melanin production was calculated by normalizing the total melanin values with protein content (μg of protein/of µL cell lysate).

### 4.5. SA-β-Gal Staining

Cells were seeded at the same density in 6-well plates at densities of 2 × 10^5^ cells/well. SA-β-Gal staining was performed using the SA-β-Gal staining kit (Cell Signaling Technology) following the manufacturer’s protocol. SA-β-Gal is a commonly used senescence biomarker and can be detected at pH 6.0 because senescent cells express a high level of lysosomal β-Gal. Image analysis was performed using the Image J software (Available at: http://reb.info.nih.ogv/ij/).

### 4.6. Western Blotting

Cells were lysed in protein lysis buffer (Intron, Seongnam, Korea) and centrifuged at 13,000 rpm for 10 min. Protein concentrations were determined using a bicinchoninic acid protein assay kit. Next, 20 μg of protein per lane was separated by SDS-polyacrylamide gel electrophoresis and blotted onto nitrocellulose membranes, which were then blocked with 5% skim milk in Tris-buffered saline containing 0.5% Tween 20. The blots were incubated with the appropriate primary antibodies at a dilution of 1:1000, and then further incubated with a horseradish peroxidase-conjugated secondary antibody following the manufacturer’s data sheet. The bound antibodies were detected using an enhanced chemiluminescence kit (Pierce, Rockford, IL, USA).

### 4.7. Real-Time RT-PCR

Total cellular RNA was extracted using the Favor PrepTM Total RNA purification mini according to the manufacturer’s instructions (Favorgen, Ping Tung, Taiwan), and the single-stranded complementary DNA (cDNA) was synthesized from 2 μg of total RNA using RevertAid First Strand cDNA Synthesis kit (Thermo Scientific, Rockford, IL, USA). qRT-PCR was performed using the LightCycler^®^ 480 II machine coupled with SYBR Green chemistry (Roche Applied Science, Indianapolis, IN, USA). In terms of qRT-PCR settings, initial denaturation was performed at 95 °C for 5 min, followed by amplification at 95 °C for 10 s, 60 °C for 10 s, and 72 °C for 10 s for 45 cycles. The cDNA obtained was amplified with the following primer in Table 2. Primers specific for GAPDH was used for loading control amplifications.

### 4.8. Immunohistochemistry

Biopsies from patients were fixed in 10% buffered neutral formalin and embedded in paraffin. These specimens were cut into 4-μM sections, and serial sections were then prepared for immunohistochemistry. For antigen retrieval, the sections were autoclaved in antigen unmasking solution (Vector Laboratories, Burlingame, CA, USA) and then immunostained using the Vector Elite ABC kit (Vector Laboratories) in accordance with the manufacture’s instruction.

### 4.9. Immunofluorescence Double Staining

A skin biopsy was taken from the normal skin and from SL skin lesions. Biopsy tissues from patients were embedded in an OCT compound (Sakura Finetek USA, Inc., Torrance, CA, USA) for histological analysis. These specimens were cut into 6-μM sections. The frozen tissues were fixed in cold acetone for 10 min. After acetone removal, the tissues were washed with PBS for 10 min at room temperature. The skin tissues were blocked with normal blocking serum for 20 min and then incubated overnight with their respective primary antibodies. After washing, the skin tissues were stained with Alexa-Fluor 546-conjugated (for MART-1; Cascade Biologics) secondary antibody for 30 min. The sections were then rinsed with PBS and incubated with the second primary antibody for 2 h at room temperature. The samples were then washed and incubated with FITC-conjugated goat anti-rabbit IgG (for Rb and CCR2; Southern Biotech, Birmingham, AL, USA) secondary antibody for 30 min at room temperature. After DAPI staining, the sections were mounted and the localization of Rb, CCR2, and MART-1 was detected using a confocal laser scanning microscope (LSM 710; Carl Zeiss, Jena, Germany).

### 4.10. Statistical Analysis

The statistical significance of the differences between groups was assessed by analysis of variance, followed by the Student’s *t*-test. *p*-values < 0.05 were considered significant.

## Figures and Tables

**Table 1:** Summary of histopathological findings in 21 solar lentigo (SL) patients.

Patient	Sex	Age (Years)	Biopsy Site	Epidermal Thickness (μm)	Rete Ridge	No. of Keratinocytes (Per One Melanocyte)	Increment of Melanocytes
1	F	53	cheek	47	Normal	4	+
2	F	59	cheek	65	Elongated	10	−
3	F	58	nose	60	Flattened	4	+
4	F	66	cheek	125	Flattened	6	+
5	F	56	cheek	110	Flattened	9	−
6	F	75	cheek	55	Normal	6	+
7	F	57	cheek	35	Normal	9	−
8	F	66	temple	42	Flattened	11	−
9	F	58	temple	55	Flattened	10	−
10	F	58	nose	60	Flattened	5.5	+
11	F	60	upper eyelid	100	Normal	4.5	+
12	F	67	cheek	43	Normal	4	+
13	F	60	nose	47	Flattened	7.5	−
14	F	61	cheek	85	Normal	4.5	+
15	F	58	cheek	95	Flattened	4	+
16	F	55	cheek	70	Flattened	3	+
17	F	62	nose	45	Elongated	4	+
18	F	55	nose	40	Normal	7	−
19	F	54	cheek	35	Flattened	5	+
20	F	50	forehead	65	Elongated	10	−
21	F	64	cheek	47	Normal	3	+
mean		59.6	-	63.1	-	6.3	-
% positivity							13/21 (62%)

**Table 2:** Primer sets used for real-time RT-PCR.

Name	Forward	Reverse
p53 (120 b.p)	TGAGGTTGGCTCTGACTGTA	TTACCACTGGAGTCTTCCAG
p21 (113 b.p)	ACAGCAGAGGAAGACCATGTGGACC	CGTTTTCGACCCTGAGAGTCTCCAG
PAI-1 (150 b.p)	TGAAGATCGAGGTGAACGAG	GGTCATGTTGCCTTTCCAGT
GAPDH	CCCATCACCATCTTCCAGGAG	GTTGTCATGGATGACCTTGGC

## Abbreviations

SL	solar lentigo
MCP	monocyte chemoattractant protein
UVR	ultraviolet radiation
NHMs	normal human melanocytes
NHKs	normal human keratinocytes
SASP	senescence-associated secretory phenotype
IL	interleukin
SA-β-Gal	senescence-associated beta-galactosidase
PBS	phosphate-buffered saline
ELISA	enzyme immunosorbent assay
OD	optical density
MTT	3-(4,5-dimethylthiazol-2-yl)-2,5-diphenyl tetrazolium bromide
TNF	tumor necrosis factor
LSM	laser scanning microscope
PAI-1	plasminogen activator inhibitor-1
POMC	proopiomelanocortin
CRH	corticotropin releasing hormone
ACTH	adrenocorticotropic hormone
MSH	melanocyte-stimulating hormone
